# Shape-driven deep neural networks for fast acquisition of aortic 3D pressure and velocity flow fields

**DOI:** 10.1371/journal.pcbi.1011055

**Published:** 2023-04-24

**Authors:** Endrit Pajaziti, Javier Montalt-Tordera, Claudio Capelli, Raphaël Sivera, Emilie Sauvage, Michael Quail, Silvia Schievano, Vivek Muthurangu

**Affiliations:** 1 University College London, Institution of Cardiovascular Science, London, United Kingdom; 2 Great Ormond Street Hospital, Cardiac Unit, London, United Kingdom; University of Michigan, UNITED STATES

## Abstract

Computational fluid dynamics (CFD) can be used to simulate vascular haemodynamics and analyse potential treatment options. CFD has shown to be beneficial in improving patient outcomes. However, the implementation of CFD for routine clinical use is yet to be realised. Barriers for CFD include high computational resources, specialist experience needed for designing simulation set-ups, and long processing times. The aim of this study was to explore the use of machine learning (ML) to replicate conventional aortic CFD with automatic and fast regression models. Data used to train/test the model consisted of 3,000 CFD simulations performed on synthetically generated 3D aortic shapes. These subjects were generated from a statistical shape model (SSM) built on real patient-specific aortas (N = 67). Inference performed on 200 test shapes resulted in average errors of 6.01% ±3.12 SD and 3.99% ±0.93 SD for pressure and velocity, respectively. Our ML-based models performed CFD in ∼0.075 seconds (4,000x faster than the solver). This proof-of-concept study shows that results from conventional vascular CFD can be reproduced using ML at a much faster rate, in an automatic process, and with reasonable accuracy.

## Author summary

In the clinical management of pediatric disease (namely congenital heart defects), the indications for ‘when’ and ‘how’ to intervene are often unclear. It has been found that haemodynamic modelling tools such as computational fluid dynamics (CFD) simulations are useful in assisting clinicians and surgeons to better understand patient conditions and establish any potential risk factors. While this tool remains useful in a research capacity, its separation from clinical settings is an ongoing hindrance which prevents the full adoption of CFD in healthcare. The translation of CFD towards clinics is a continuous challenge, due to large time, computational and human resource requirements for running simulations. The application of machine learning (ML) for exploring potential methods to transform conventional CFD into clinically-suitable models is a recent phenomenon which is gaining significant momentum.

This is a *PLOS Computational Biology* Methods paper.

## Introduction

Computational fluid dynamics (CFD) has significant potential in cardiovascular settings, with applications including modelling of complex flow patterns and non-invasive estimation of vascular pressure [1]. Importantly, several studies have validated CFD measurements against conventional clinical methods such as 4D cardiovascular magnetic resonance (CMR) imaging and catheter-based pressure measurements [2, 3]. A major strength of CFD lies in situations where it is difficult to acquire data with conventional clinical means. For example, studies have shown CFD can be used to assess the haemodynamic response to exercise as a non-invasive alternative to cardiac catheterisation with exercise or pharmacological stress [4]. Another example is the use of CFD for predicting the haemodynamic response to specific interventions, such as aortic stenting in patients with coarctation [5]. Furthermore, there is increasing interest in exploring CFD-derived metrics for risk stratification of patients. For example, Qiu et al. demonstrated that CFD derived helical flow patterns were associated with significantly increased risk of abdominal aortic aneurysm rupture [6]. Thus, integration of CFD into clinical settings may have important ramifications when used: (i) for detailed assessment of patient-specific haemodynamics in response to stress or interventions, and (ii) to estimate CFD-derived indices for supporting clinical decision-making and risk-stratification.

Despite potential benefits, CFD is still not integrated into routine clinical practice. This is mainly due to long computation times, the requirement for large amounts of processing power, and the need for an experienced engineer to set up simulations correctly [7]. Recently, machine learning (ML) models have been successful in replacing time-consuming or computationally intensive tasks, such as medical image segmentation [8]. Similarly, the use of ML for speeding up cardiac CFD tasks is also becoming increasingly studied [9, 10]. The main challenges of training ML models using CFD data include: (i) poor availability of clinical data, (ii) unstructured meshes without point correspondence, and (iii) large meshes and resultant CFD flow fields. To overcome these problems, we used statistical shape modelling and dimensionality reduction techniques to produce dimensionality-reduced representations of both aortic shape and flow fields, and to enable creation of large amounts of synthetic training data [11, 12]. This approach relies on the fact that in the past it has been show haemodynamic flow structures can be regressed from shape features [13].

Our primary aim was to show the feasibility of a shape-driven ML model for the accurate estimation of 3D CFD flow fields in a population of aortas with challenging shape features. The main aims of this study were: (i) to create a large synthetic cohort of 3D aortas based on real clinical cases with complex anatomies, (ii) to train ML models to predict aortic pressure and velocity fields by representing unstructured/large data types with low-dimensional vectors, and (iii) to compare results between our fast/automatic ML-based CFD solution and our conventional CFD method on both synthetic and real aortic cases.

## Materials and methods

### Statistical shape modelling

**Fig 1:**
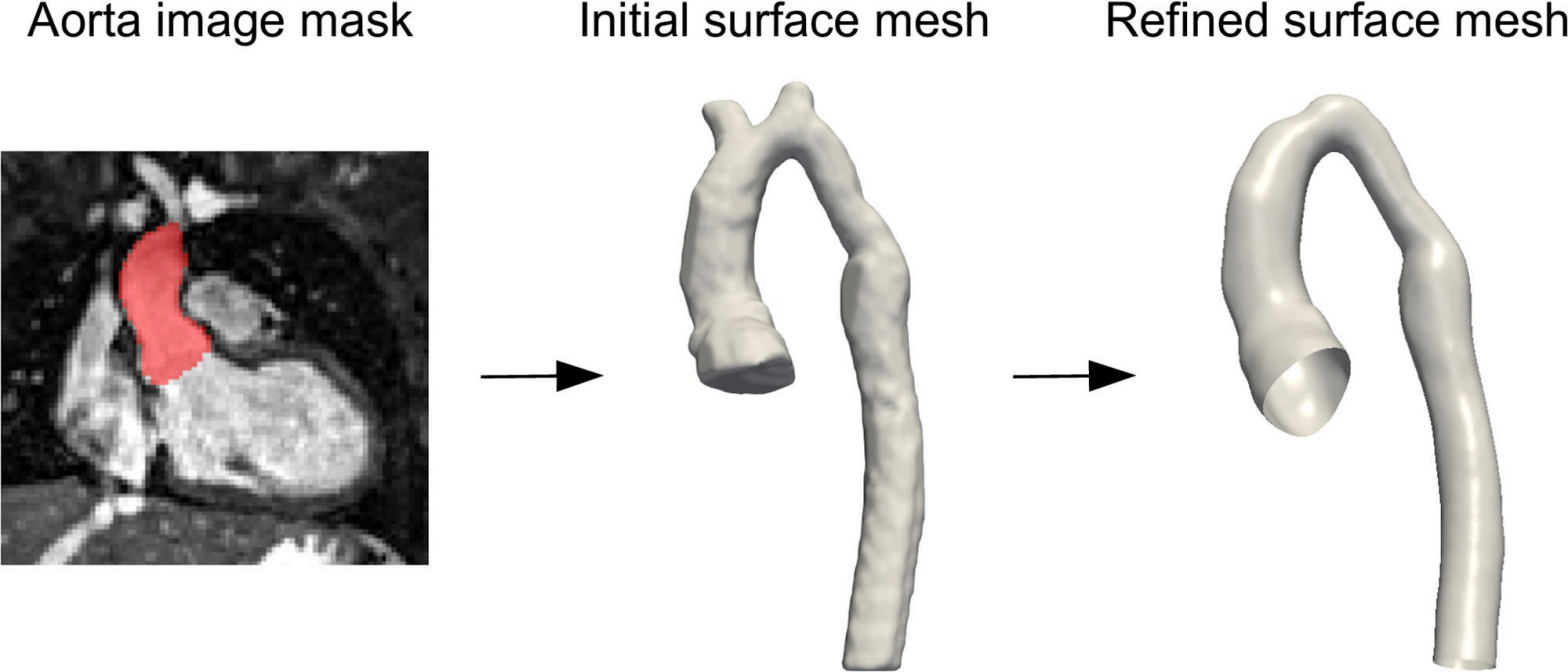
Segmentation and mesh-preprocessing pipeline. The aorta segmentation of each subject is re-meshed and smoothed in an automatic pipeline. This is followed by clipping of the inlets/outlets and head & neck vessels.

The dataset used for development of the statistical shape model (SSM) consisted of cardiac and respiratory gated steady state free precession CMR images (N = 67) from patients previously diagnosed with coarctation of the aorta (CoA). All patients were post-surgical repair, asymptomatic and underwent CMR imaging at a mean age of 22.4 ±6.2 years. The use of retrospectively collected data was approved by the local research ethics committee, and written consent was obtained from all subjects/guardians (Ref: 06/Q0508/124). Images for each subject were segmented and converted into surface meshes (Fig 1). This was followed by remeshing and smoothing using functions from the vascular modelling toolkit (VMTK) [14]. All geometries were aligned in the same local space and orientation through rigid registration using an iterative closest point algorithm in VMTK [15]. This ensured that shape modelling was not affected by any spatial misalignment. Surfaces were manually clipped above the aortic root for the inlet, and at the diaphragm for the outlet. An SSM was then built using these clipped aortic surfaces, using an approach previously described by Bruse et al [16].

**Fig 2:**
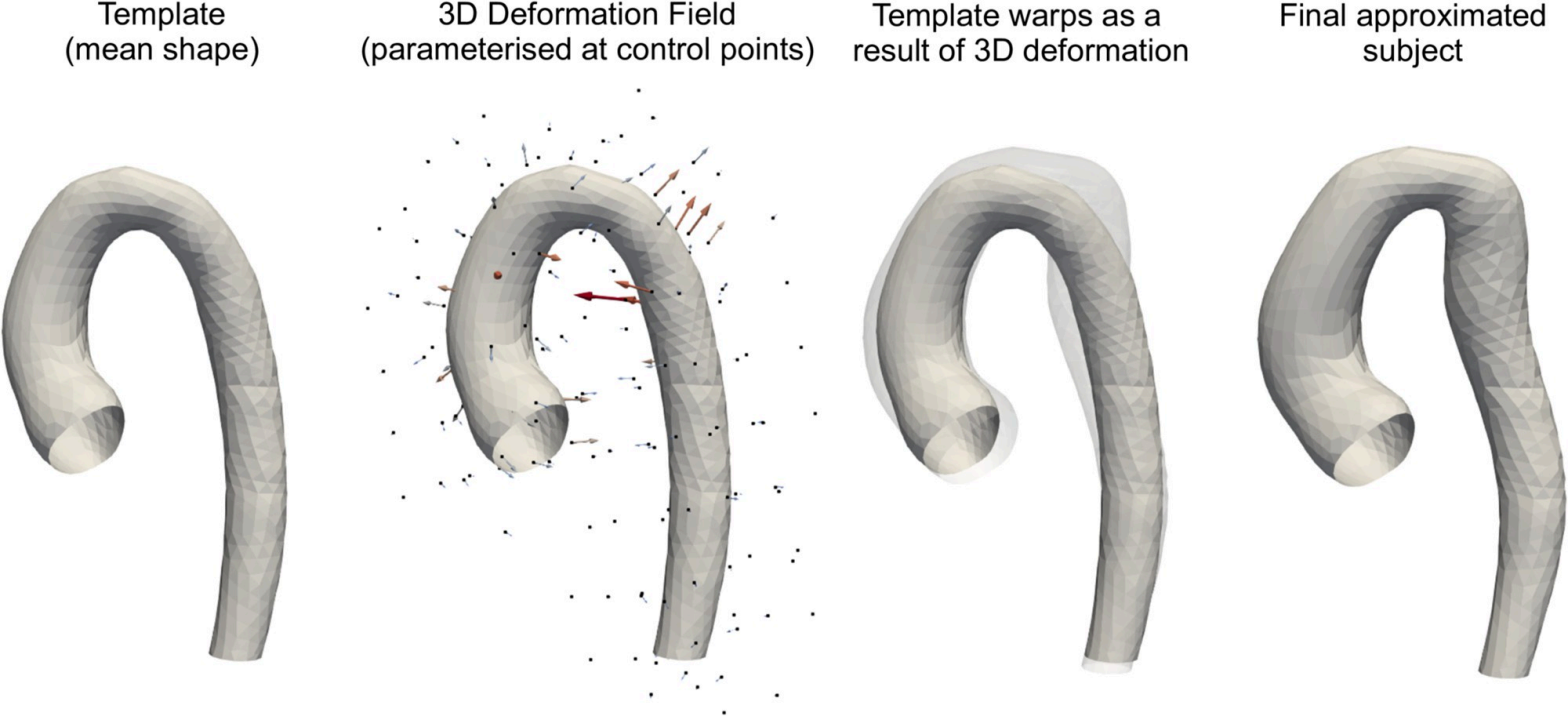
Mesh registration with SSM. An example aortic shape approximation using our SSM is shown. Individual surface or volumes can be reconstructed using a mean aortic shape and applied deformation field initialised on a set of control points (n = 172).

The package Deformetrica 4 was used to build the SSM [17]. First, an average aortic shape (surface template) was computed, containing 2,541 nodes (Fig 2). A volume template was also created by meshing the surface template with tetrahedral elements (29,000 nodes). Each subject could then be described as a non-linear deformation of 3D ambient space, relative to the template (Fig 2). In this case, each deformation is fully parameterised by a paired set of 3D control points (*q*_*i*_)_*i*=1,…*n*_ and 3D momenta vectors (*μ*_*i*_)_*i*=1,…*n*_ using a Gaussian kernel of width *σ* which we set as 10 mm. The number and location of control points were optimised (n = 172) by initialising the model with a high resolution control point grid (n = 500) and truncating points which were observed to have little influence on the deformation (low variance). The final computed 3D deformations for a given subject were represented by a deformation vector with 516 coefficients—the number of control points (172) multiplied by the number of deformation directions (3). The deformation vectors for the whole population were collected in the 2D matrix M [67, 516], which was decomposed using principal component analysis (PCA) in order to identify low-dimensional deformations which account for most of the variance. This required standardisation for each column in M (i.e. removal of the mean and scaling to unit variance). Following this, singular value decomposition (SVD) was performed, with M being decomposed into three matrices: M = USV^T^. The V transpose matrix [516, 516] contained the principal component axes or PCA modes, S is a rectangular diagonal matrix [67, 516] where the singular values on the diagonal can be used to calculate the variance explained by the associated PCA modes, and the matrix product US [67, 516] contains the projection (weights) of each subject onto each PCA mode. It was found that the first 35 PCA modes were capable of approximating 99% of the variance in M. This meant that specific aortic shapes could be represented by a lower-dimensional deformation vector with 35 coefficients rather than 516 (almost 15 times reduction in the size).

Using the SSM, new synthetic aortic shapes could then be created using synthetic lower dimensional deformation vectors. Specifically, each of the 35 coefficients in a synthetic vector was generated by randomly sampling a Gaussian distribution (within 2 standard deviations) based on the distribution of weights in the US matrix. Following concatenation of all the lower dimensional deformation vectors, the matrix X [3000, 35] was transformed into a matrix L [3000, 516] by matrix multiplication (L = XSV^T^), thus reversing PCA and mapping the matrix X onto the original axes. Standardisation was then reversed in all columns of L using the previously computed standard deviations and means in the M matrix. The deformation matrix L was then reshaped into a 3D momenta matrix [3000, 172, 3] and applied onto the aortic surface and volume template using Deformetrica, thus generating a surface and volume mesh for each new synthetic subject. Since all new meshes are derived from the same template, all synthetic aortas contained the same number of nodes/elements. Additionally, nodes can be thought to be lying within spatially correspondent locations within each aorta (see S1 Fig). This was vital for enabling the dimensionality reduction of derived flow fields, as described in later sections.

The new synthetic population (n = 3000) was compared to the original population (n = 67) by computing geometric properties of the shapes based on a centreline approach. Mean centreline lengths and diameters were computed. Mean torsion is used to express how sharply the centreline is twisting in space. The parameter tortuosity describes the length ratio between the centreline and a rectilinear line between the endpoints. All parameters were computed using implementations within VMTK, as described by Piccinelli et al. [18].

### Computational fluid dynamics pipeline

**Fig 3:**
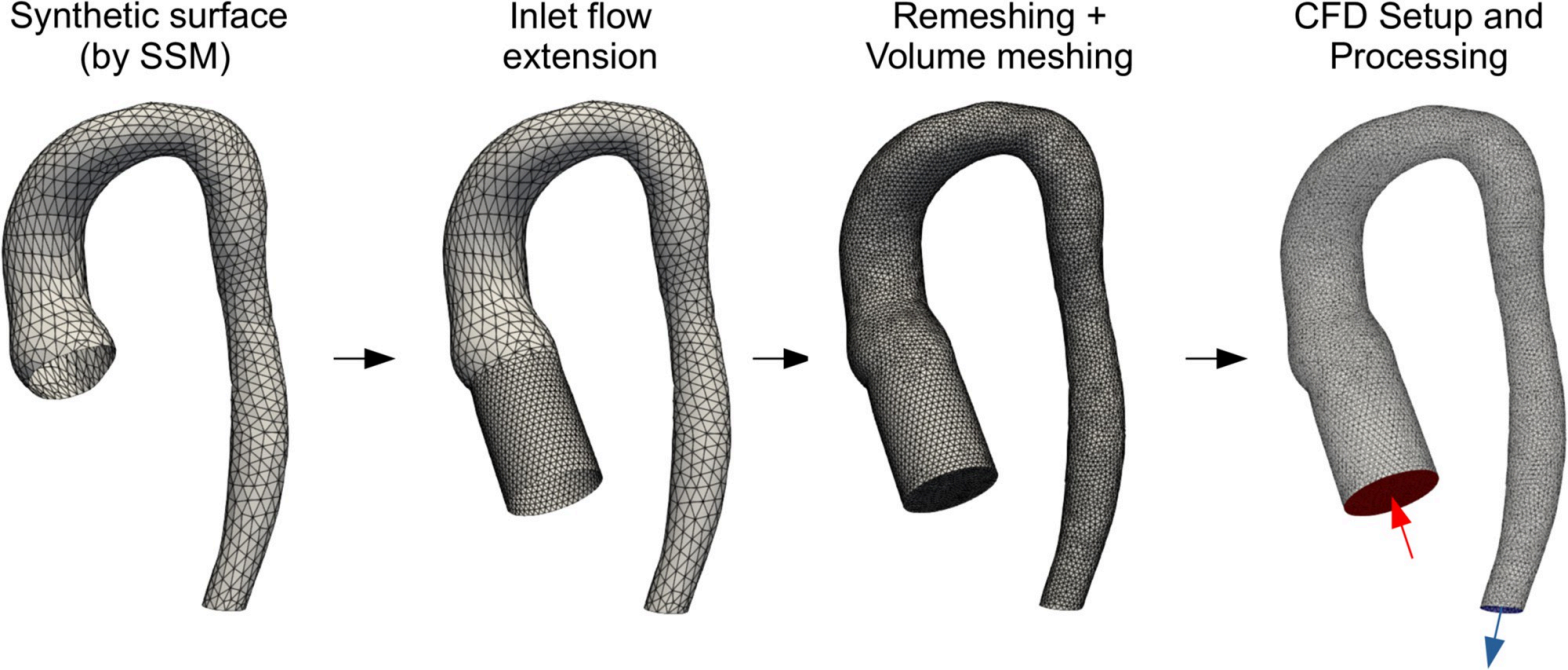
CFD Pipeline. Surfaces have flow extensions added before volume meshing. The same simulation set-up is applied to the final volume mesh for each synthetic subject.

Volume meshes previously generated with the SSM were unsuitable for CFD computation. This was primarily because low mesh skewness could not be guaranteed, and remeshing was not an option since nodes were to be preserved in order to maintain point correspondence (see S1 Fig). Therefore, separate meshes solely for CFD computation were built, starting from the surface of each aorta. Firstly, each surface was extended by 40mm at the inlet (Fig 3). This was done to produce a flat and circular inlet upon which a velocity profile could be uniformly applied. Extending the inlet further than 40mm was avoided in order to reduce the likelihood of surface self-intersection. Following this, volume meshing with tetrahedral elements was performed [19] (∼400,000 cells on average). Element/node counts were deemed to be in satisfactory ranges after a mesh sensitivity analysis (see S2 Fig).

CFD (Fluent, Ansys Technologies) was performed on all 3,000 synthetic cases. The same boundary conditions were applied to each simulation, as part of adopting a simple model which would reliably converge for all subjects. Laminar, steady-state flow conditions were enforced. An inlet velocity of 1.3 m/s, corresponding to an average ascending aortic flow rate at peak systole, was set [20, 21]. A velocity boundary condition was preferred to a volumetric flow rate, since it is invariant to any differences in inlet surface area between subjects. Outlet gauge pressure was fixed at 0 Pa. Standard non-slip conditions were applied at the wall, and the fluid was assumed to be Newtonian with density and dynamic viscosity equal to 1,060 kg/m^3^ and 0.004 Pa⋅s, respectively [22]. The set-up and simulation of all 3,000 cases was fully automated.

### Machine learning

#### Data interpolation and principal component analysis

**Fig 4:**
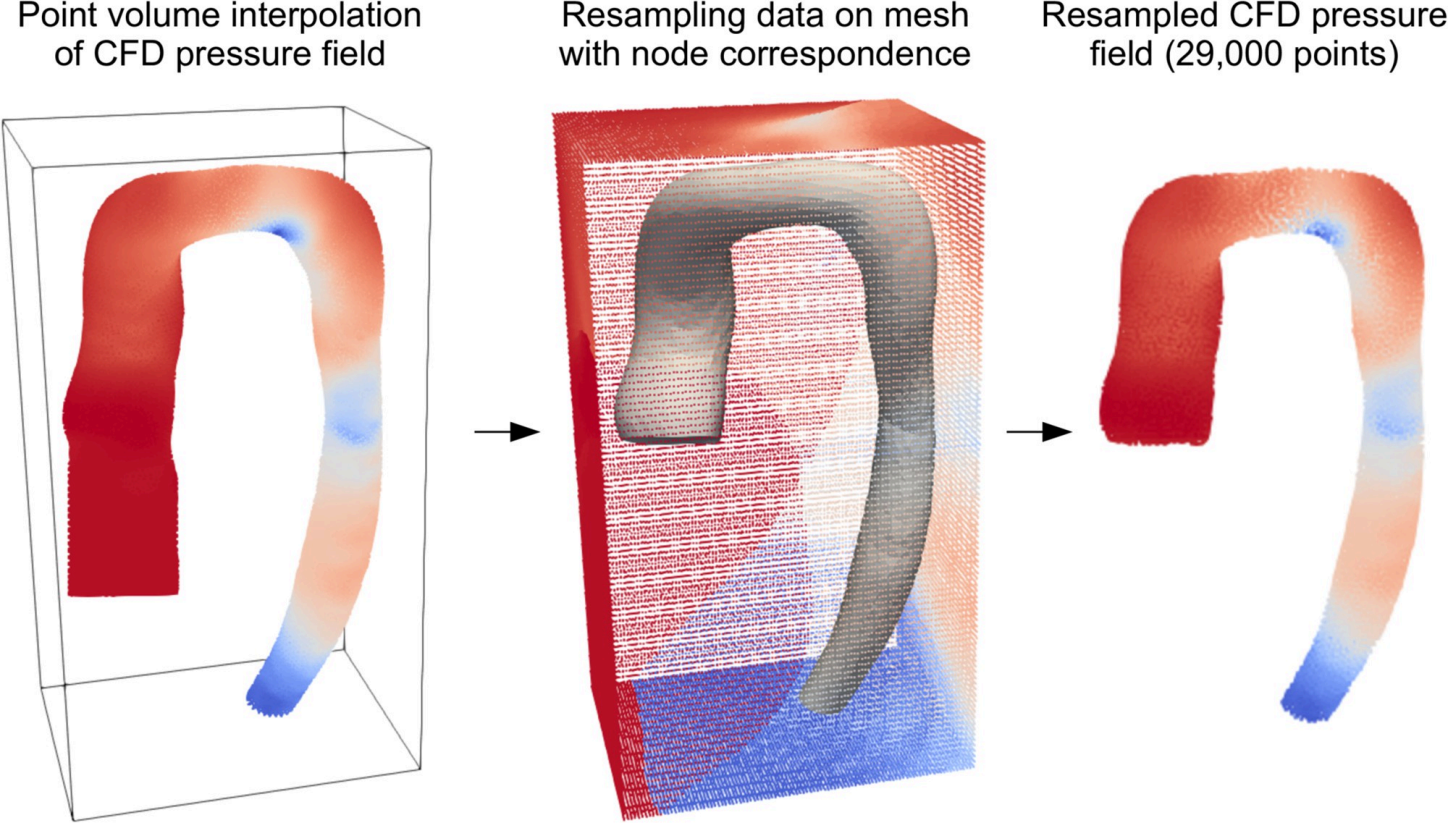
CFD data interpolation. CFD results are interpolated onto a point-correspondence mesh (generated by the SSM), thus restoring node concordance.

As CFD was performed on large unstructured meshes with inconsistent numbers of nodes/elements between cases, point correspondence had to be restored prior to PCA-based dimensionality reduction of the flow fields (needed for easier model training). This was done using the volume meshes previously generated with the SSM by ‘shooting’ on the template. Since each of these ‘SSM volume meshes’ inherited its properties from the template, relative nodal positions were preserved. Consequently, pressure/velocity data for all subjects were re-sampled from unstructured CFD meshes onto SSM volume meshes using a Voronoi kernel (padding of 5 mm, grid resolution of 1,000,000 voxels) in the software Paraview (Fig 4). This resulted in 3,000 newly resampled pressure/velocity fields, with all subjects containing 29,000 nodes in point correspondence. The data was concatenated into a feature vector and PCA was applied to reduce dimensionality. We aimed to capture 99% of variance with as few PCA modes as possible.

#### Deep neural network architecture

**Fig 5:**
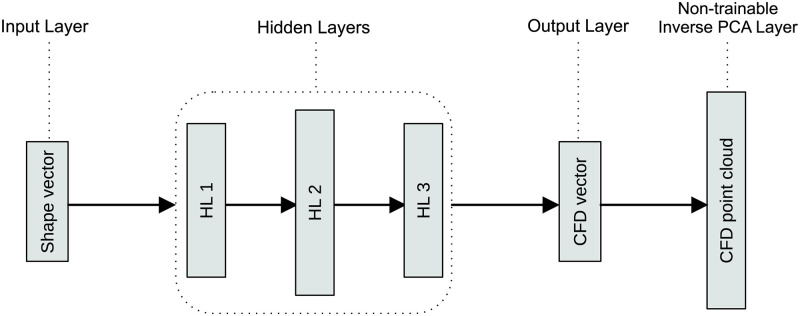
DNN general architecture. The general sequential, fully-connected DNN set-up used to build both pressure and velocity predictors (’CFD vector’ can be either pressure or velocity PCA vectors).

The architecture we adopted was a standard sequential, fully-connected deep neural network (DNN) with independent networks for pressure and velocity. The input for the model was the lower dimensional deformation vector, which is also referred to as a ‘shape vector’. The outputs of the trainable part of the model were the pressure/velocity PCA scores (reduced order CFD field), referred to as a ‘pressure/velocity vector’. A non-trainable inverse PCA layer (implemented in Keras using a lambda layer) serves to reconstruct the pressure/velocity vector into the full 3D flow field with 29,000 nodes (see Fig 5). Rectified linear units (ReLU) were used in each hidden layer. Linear activation functions were set at the output. Model implementation was done using Keras and TensorFlow 2.0.

#### Deep neural network training

Models were built separately for predicting the static pressure and the velocity-magnitude. The loss function used for training was the mean absolute error (MAE), computed on the entire 3D flow field (i.e. after inverse PCA) rather than on the output pressure/velocity vector. This provides a more granular measure of error and effectively weights the importance of each PCA mode in the network according to the amount of variance it explains. Model optimisation was carried out using the Adam optimiser [23]. The training dataset was composed of 2800 randomly selected subjects, with the remaining (200) going into the test set.

Hyperparameter tuning was conducted using 5-fold cross-validation to find model settings which generalise well for unseen data. For this, the training dataset was split into 5 groups (580 subjects each). The model was trained and tested five times, with each group taking turns to act as the validation set while the remaining four were used for training. The average validation error for all five training runs was used to represent the performance of that model. This process was repeated for 1,000 model configurations, which were sampled using a tree-structured Parzen estimator (TPE) algorithm [24]. The number of hidden layers, number of hidden layer neurons and initial learning rate were all explored. Batch size and epochs were set at 32 and 50, respectively. Hyperband pruning was used to terminate early training rounds if the model was deemed to be poorly fitting the validation data. After completing hyperparameter tuning, the best model was retrained for 1000 epochs using a larger portion of the training set (2,600 subjects) with 200 remaining for validation. Model training lasted 1,000 epochs and training/validation loss was monitored to prevent overfitting. Model training was performed using an Nvidia GTX 1080Ti graphics card.

#### Model evaluation

Once trained, the model was evaluated on the test set of synthetic aortas (n = 200). Absolute errors were computed for every node in all test cases by comparing the prediction value (ML) to the ground-truth value (CFD). Errors were then normalised according to subject CFD data range, as detailed in Liang et al. [11]. Normalisation was necessary to enable direct comparison between individual cases and also between CFD metrics, since pressure/velocity ranges widely differed per subject. Eq 1 details how normalised absolute error (NAE) is computed for either pressure or velocity at a node *i*, belonging to a subject *j*. *True*_*i*,*j*_ is the CFD nodal pressure/velocity value. *Pred*_*i*,*j*_ is the ML nodal pressure/velocity value. *Range*(*True*_*j*_) is the difference between the maximum and minimum values in the CFD flow field for subject *j*.

$$\begin{array}{c} NAE\left(i,\;j\right)=\frac{\left|True_{i,\;j}\;-\;Pred_{i,\;j}\right|}{Range(True_{j})}\times 100\% \end{array}$$ (1)

Mean node errors (MNAE_N_) were computed by averaging NAE values across the population for each node (n = 29,000). These were then plotted on the template mesh points in order to better visualise the magnitude of these errors with respect to their location. However, since NAE values are absolute errors, this provides no insight regarding any systematic over or under-estimation during model inference. Therefore, a Bland-Altman plot was used to examine the bias and limits of agreement of the pressure and velocity DNNs. This was done for the overall aorta and for three separate regions; ascending aorta, transverse arch and descending aorta (anatomically defined).

Mean subject errors (MNAE_S_) were computed by averaging NAE values in each subject (n = 200). Cases with the best, median and worse mean subject error values were compared. A single population error for both pressure and velocity was given by averaging all MNAE_S_ values. The relationship between shape mode scores and subject error (MNAE_S_) was investigated with scatter plots and assessed using Pearson R coefficients and p values (*p* = 0.05 considered significant).

In addition to evaluation of the models on the test set (n = 200), the models were also tested on real, patient-specific aortas with previously repaired CoA (n = 10), completely unseen from the SSM and the DNN. This was done in order to validate the robustness of the models for inferring accurate flow fields on real subjects outside our synthetic training/testing sets. CMR images of each patient were segmented. Surfaces were approximated by non-rigid registration (applying deformations on the template) using the SSM. Deformation matrices for each case were decomposed into PCA shape vectors and passed as inputs into the DNN models. Pressure and velocity-magnitude fields were inferred for each subject. Following this, all ten predictions were compared to CFD flow fields computed using both SSM derived geometries and real geometries.

**Fig 6:**
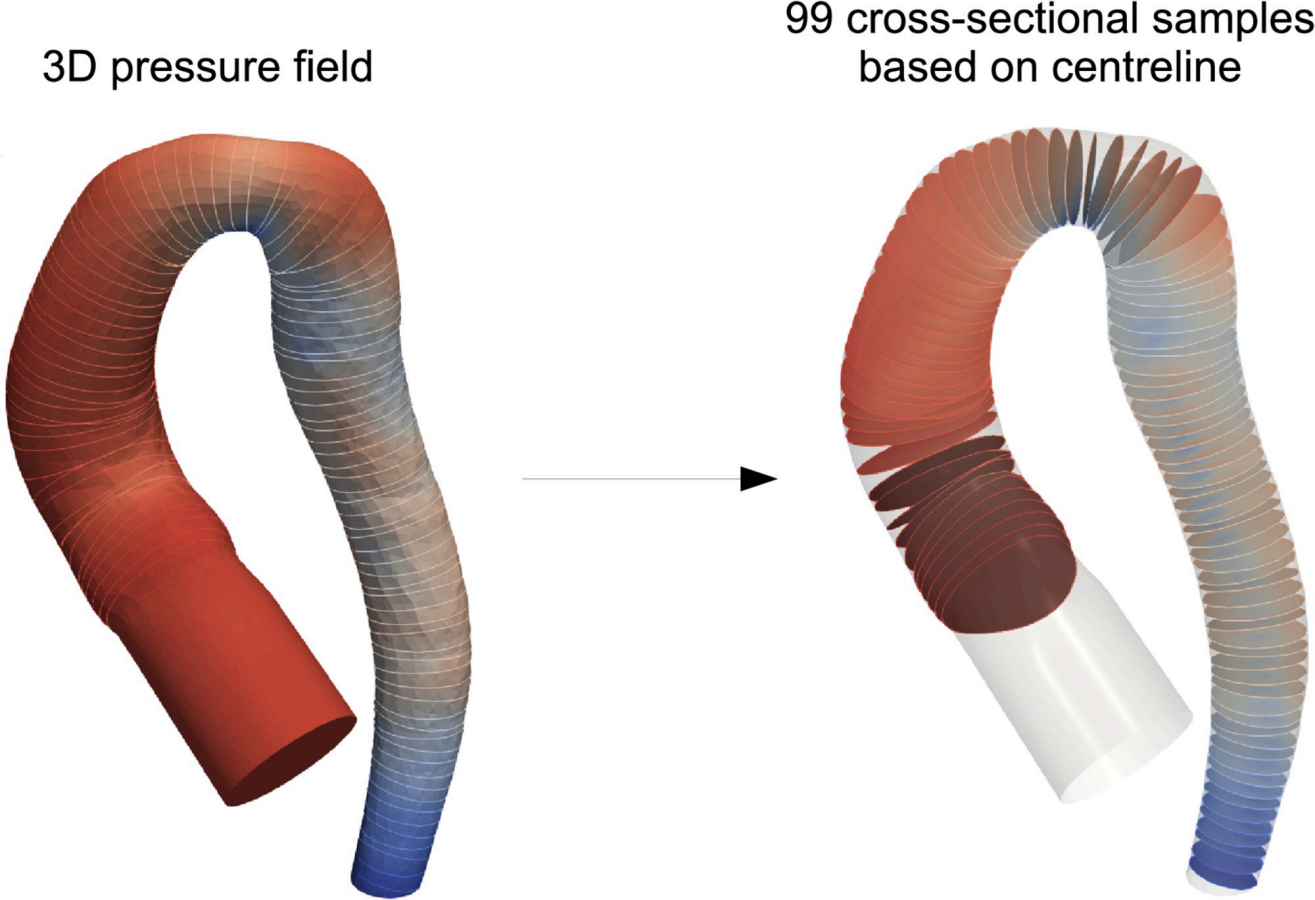
CFD gradient extraction method. Using subject centrelines, 99 plane-averaged pressure or velocity points along the length of the aorta are extracted by sampling the 3D flow fields. The origin is always the aortic root (excluding the extension).

Comparison between ML and SSM derived flow fields were made using MNAE_S_ as previously described. However, it was not possible to compare the predicted and real CFD flow fields with node-based metrics due to the lack of node-to-node correspondence and exact surface matching. Therefore, we used a gradient-based approach to enable direct comparison of pressure/velocity flow fields without shape correspondence. Subject centrelines were used to calculate plane-averaged pressure/velocity gradients at 99 locations over the length of the aorta (Fig 6). To compare gradients, the Fréchet distance (FD) was used. The FD is a measure of similarity between two point-sets of curves, taking into account the location and ordering of the curve coordinates. Intuitively, it can be thought of as the shortest possible distance between two observers traversing different paths while remaining connected. An advantage of using the FD is that it does not neglect sharp spikes or differences between gradients, which some other metrics may diminish through averaging. Additionally, since FD is not a percentage error, it does not emphasise errors where values are close to zero (such as at the very end of the descending aorta in pressure flow fields). An algorithmic implementation for computing FD as described by Eiter et al. was used to calculate this metric [25].

## Results

### Statistical shape modelling

**Fig 7:**
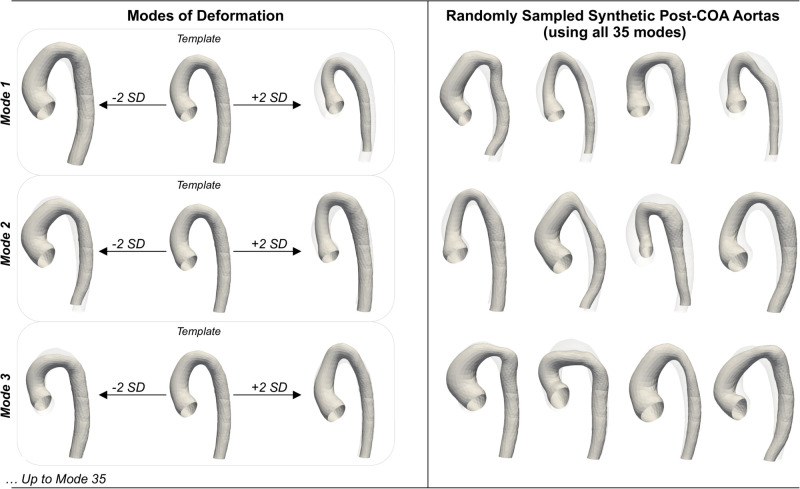
Modes of deformation. Left: first three modes of deformation from the SSM (SD = standard deviation). Right: examples of synthetic post-CoA aortas from the test set (using combinations of all 35 shape modes).

PCA decomposition was performed on the deformation matrices (momenta) computed by statistical shape modelling. The first, second and third PCA modes captured 29.8%, 13.2% and 10.1% (total 53.1%) of the variability, respectively. Mode 1 relates to overall vessel size, mode 2 relates to ascending arch angulation/diameter, and mode 3 describes rounded versus triangular arches. After PCA decomposition, 99% of the variance in the momenta could be represented with the first 35 modes. Some examples of the 3,000 synthetic subjects produced by randomly sampling and combining 35 PCA mode scores are shown in Fig 7. Anatomical characteristics of the synthetic aortas (length, diameter, tortuosity and torsion) were found to be statistically similar to those of the real patient cohort (see Table 1).

**Table 1:** Comparison of aorta dimensions between original real cohort (n = 67) and synthetic cohort (n = 3000).

	Mean diameter (mm)	Length (mm)	Tortuosity	Mean torsion
Real cohort average (n = 67)	19.74 ±1.29	257.3 ±29.88	2.21 ±0.39	0.0044 ±0.061
Synthetic cohort average (n = 3000)	20.12 ±1.32	258.5 ±23.08	2.19 ±0.35	0.0012 ±0.053
*p* value (Welch’s t-test)	0.25	0.75	0.74	0.65

### Training data

After CFD was computed on all cases (n = 3000), values were interpolated from high resolution meshes onto lower-resolution grids in point correspondence. The mean loss in accuracy due to interpolation was found to be 0.056% ±0.027 and 0.849% ±0.247 for pressure and velocity-magnitude, respectively. This was computed by calculating the mean percentage error in centreline pressure and velocity gradients for all cases (n = 3000) and averaging the results. The data post-interpolation was used as the ‘ground-truth’ training and testing sets.

PCA decomposition of the pressure and velocity training data matrices ([2800, 29000] each) was then performed, following standardisation. After PCA decomposition, 99% of the standardised pressure variance could be captured with 20 modes. Only 87% of the standardised velocity variance could be captured with 55 modes, and it was felt that adding more modes to capture greater variance was not feasible due to massively diminishing returns. Subject errors resulting from PCA decomposition were tested on the 200 test cases (unseen by the PCA model). Average MNAE_S_ in pressure and velocity fields were found to be 1.46% ± 0.59 SD and 2.70% ±0.49 SD. The reconstructed test cases with the highest MNAE_S_ for pressure and velocity (4.32% and 4.93%, respectively) are shown in S3 Fig.

### Model architecture

The model input layer size was set at 35 (number of shape modes). Output layer sizes were set at 20 and 55 for pressure and velocity, respectively (number of pressure/velocity modes). Hyperparameter tuning using cross-validation was performed 1,000 times to search for the optimal learning rate, number of neurons and number of layers, with tuning taking ∼4 hours per model. Pressure and velocity model architectures as a result of the optimisation process are shown in S4 Fig.

### Model predictive performance

Pressure and velocity-magnitude fields were computed on the test set (n = 200) using the trained DNNs. Inference took 0.075 seconds per subject for both DNNs. In comparison, conventional CFD took ∼5 minutes on average for convergence, demonstrating an approximate 4,000x speed-up with ML.

#### Node errors

**Fig 8:**
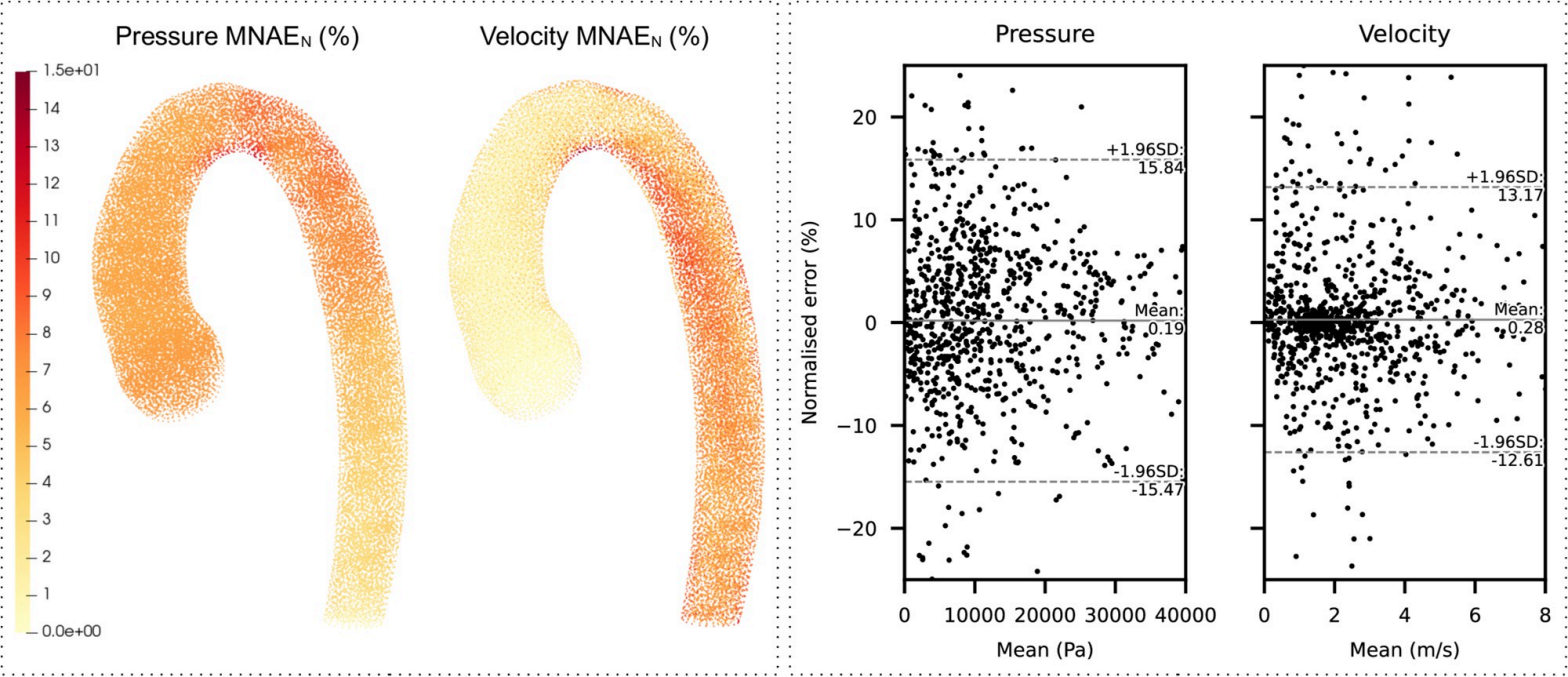
Nodal error analysis. Left: Distribution of mean nodal errors (MNAE_N_), computed on the test set (n = 200). Errors are absolute values and are projected on the template aorta. Right: Bland-Altman plots for the overall aorta. Normalised error (%) refers to the NAE of each node in every test case, without taking the absolute value (n = 5,800,000). Only 1,000 randomly selected points were drawn to improve graph readability.

Average node prediction errors (MNAE_N_) were computed for all nodes (n = 29,000). Fig 8 (left) shows these values projected onto the template (average position of the nodes), allowing for the locations of the highest absolute errors to be assessed. Pressure errors were observed to be lower in the descending aorta, with the highest errors situated in the transverse arch. Velocity errors were notably more prevalent in the underside of the arch and descending aorta. The maximum MNAE_N_ was observed to be 12.46% and 14.86% for pressure and velocity, respectively.

Bland-Altman analysis showed negligible prediction biases for the overall aorta (Fig 8, right) and within selected regions (S5 Fig). Bland-Altman biases were found to be 0.19% and 0.28% for pressure and velocity, respectively. The limits of agreement were found to be marginally wider for pressure when compared to velocity (15.65% vs 12.89%, respectively).

#### Subject errors

**Fig 9:**
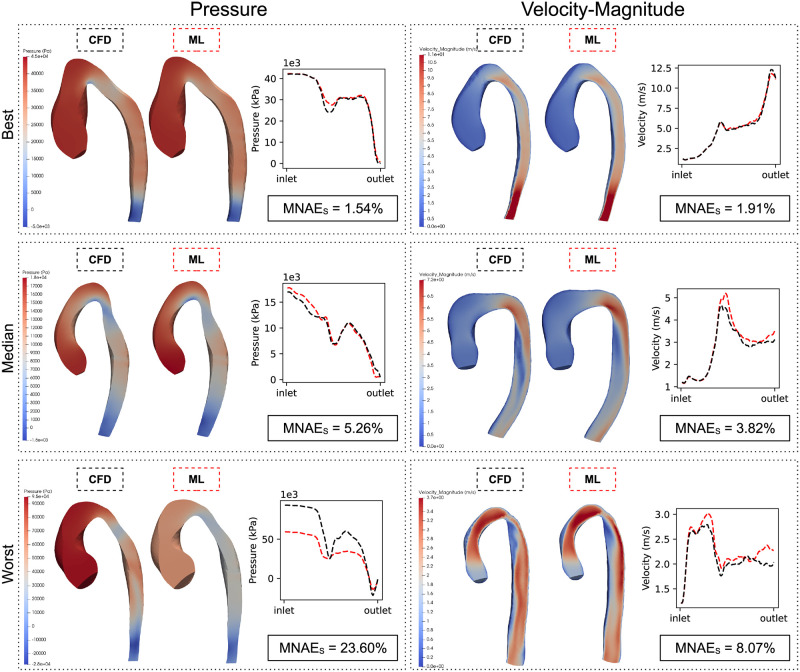
Best, median and worst test-set predictions. Comparisons between ground truth (CFD) and predicted (ML) in the test set (n = 200). Best, median and worst cases for both pressure and velocity-magnitude are shown, ranked using the mean node-to-node error (MNAE_S_). Pressure/velocity gradients are also displayed (black lines = CFD, red = ML).

The population error for pressure and velocity was 6.01 ±3.12% SD and 3.99 ±0.93% SD, respectively. The test cases with the best, median and worst subject error (MNAE_S_) are shown in Fig 9 with corresponding pressure/velocity gradients. The maximum MNAE_S_ for pressure and velocity were found to be 23.60% and 8.07%, respectively. The minimum MNAE_S_ for pressure and velocity were found to be 1.54% and 1.91%, respectively.

**Fig 10:**
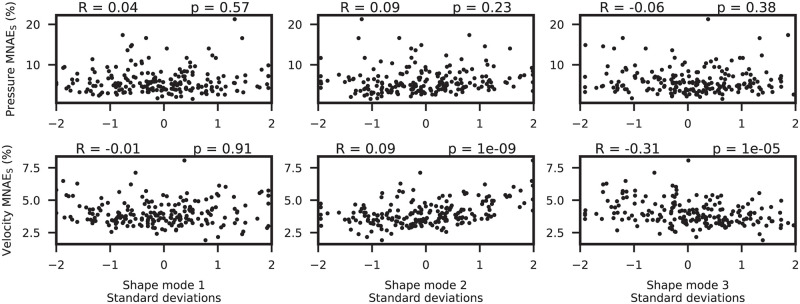
Shape modes vs. ML error. Scatter plot comparing the shape PCA mode values against subject error (MNAE_S_) in the test set (n = 200). Pearson R coefficients and p-values were computed for each subplot.

The relationship between pressure/velocity MNAE_S_ and shape mode coefficients is presented in Fig 10. The second and third mode showed statistically significant correlations with velocity prediction MNAE_S_, with the third shape mode showing the highest correlation (R=−0.31). No significant correlations were found between any other shape mode and MNAE_S_.

### Validation with real patient data

Coefficients for the first 10 shape modes for all new subjects (n = 10) were found to lie within the max-min ranges of the original PCA shape modes (n = 67, see S6 Fig). The average prediction error using the MNAE_S_ metric (ML vs CFD computed on SSM meshes) was 10.19% ±10.41 and 4.47% ±1.18 for pressure and velocity respectively. This corresponds to an increase in mean subject error by 4.18% for pressure and 0.48% for velocity, when compared to the population error in the test-set of synthetic cases (n = 200).

**Fig 11:**
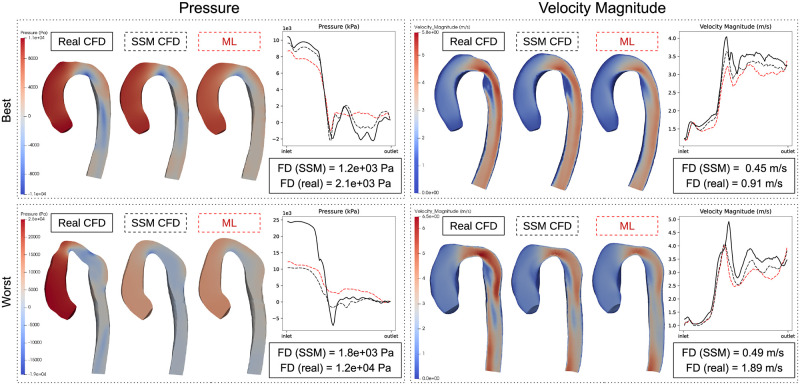
Testing on prospective data. Best and worst pressure and velocity predictions on the real patient test cohort (n = 10). FD (SSM) is the error between the predicted (red) and SSM CFD (dashed black) gradients. FD (real) is the error between the predicted (red) and true CFD (solid black) gradients.

Due to the lack of point correspondence and surface matching between the real and SSM shapes, Frechet distance of pressure and velocity gradients were used to compare the ML CFD results with the patient CFD data (computed using both SSM and real geometries). The FD computed between the ML and the CFD (SSM) gradients corresponded to the prediction error arising solely from the ML model (FD SSM). The FD computed between the ML and the CFD (real shape) gradients corresponds to the total error between the ML prediction and true CFD (FD real). The subjects with the best and worst FD (real) for pressure and velocity are shown in Fig 11. The mean FD SSM was 1680 ±629 Pa for pressure and 0.47 ±0.17 m/s for velocity. This compares to 4583 ±3210 Pa for pressure and 1.30 ±0.37 m/s for velocity between the ML and real-CFD (FD real).

## Discussion

The main findings of this study were: i) statistical shape models (SSMs) and PCA are suitable for creating synthetic training data and dimensionality-reduced representations of 3D shape and flow, ii) DNNs based on these dimensionality-reduced representations can predict pressure and velocity fields with high accuracy.

### Synthetic data generation and dimensionality reduction

A key element of our approach was to use an SSM and PCA to both generate a synthetic training dataset (n = 3,000), and to parameterise aortic shape/flow fields for simplifying DNN model training. We demonstrated that volumetric meshes generated by our SSM were suitable for forcing point correspondence in our dataset through interpolation of 3D aortic CFD flow fields. It was also shown that less than 60 PCA modes were needed to capture the majority of variance within aortic shape deformations and 3D pressure/velocity fields. Mean PCA reconstruction errors of pressure/velocity were found to be low, but not insignificant in the worst observed test-set reconstructions (S3 Fig). Geometric properties of our synthetic data were shown to be mostly close to the real cohort (Table 1). In the future, newer approaches for generating synthetic data and creating dimensionality-reduced representations of complex structures could be explored, notably deep-learning methods such as autoencoders [11] and generative adversarial networks (GANs) [26]. In some studies, autoencoders have been shown to be superior to PCA-based methods (e.g. for 3D facial surface reconstruction) [27, 28]. Additionally, GANs have shown promise for creating images of synthetic patients afflicted with CHD [29]. Finally, instead of sampling a Gaussian distribution to find combinations of shape PCA parameters, other methods for creating new DNN training data may be more appropriate, such as Latin hypercube sampling [30].

### Model performance

Our ML models were observed to predict point clouds of both pressure and velocity flow fields with good accuracy, while being approximately 4,000x faster than our conventional CFD method. Node errors for pressure were seen to be larger at the inlet, while velocity errors were more skewed towards the distal regions of the aorta (Fig 8, left). This is most likely due to the CFD boundary conditions at the inlet and outlet constraining the pressure/velocity variability at these regions. The further away from the aortic inlet or outlet, the greater the variability in velocity or pressure, respectively. It should be noted that there were no significant biases in either pressure or velocity predictions, suggesting that there were no systematic errors with the models (Fig 8, right). In testing, we found that the mean pressure subject error was slightly higher than the mean velocity error. This is despite the pressure PCA model capturing more variance than the velocity model. A possible explanation for the higher pressure errors is that the association between shape and pressure is more complex than that between shape and velocity in aortic domains. This is supported by the observation that shape modes do not correlate with pressure errors (Fig 10). Interestingly, there was a strong negative correlation between shape mode 3 and velocity error. This suggests that more ‘gothic’ aortas (characterised by a more triangular arch) were less prone to velocity prediction errors. A possible explanation may be that the gothic arch constrains downstream flow patterns (where most velocity errors occur), hence making it easier for the model to characterise flow features associated to this subset of aortic shapes.

An important element of this study was to apply trained DNN models to prospective, unseen cases in order to explore the feasibility of performing inference on real patient data (n = 10). It was shown that the average node-based prediction error (MNAE_S_) on the prospective cases did increase for pressure from 6.01% to 10.19%. Velocity errors increased only marginally in the prospective test cases (3.99% to 4.47%). This aligns with the previous observation that the relationship between shape and pressure may be more complex due to the lack of an observed correlation between the shape PCA modes and pressure prediction errors. We also compared the ML predictions to the real CFD (performed on the raw segmentation mesh) for all cases. This enabled the proportion of the total error due to the SSM and DNN to be estimated. We showed ∼60% of the total gradient error in pressure/velocity was due to the SSM, which further strengthens the argument for improved shape parameterisation.

The approach of using DNNs to model 3D aortic pressure and velocity flow fields has been described in other works [10, 11]. However, an important limitation of this data-driven approach is that flow fields cannot be assumed to satisfy the Navier-Stokes equations for incompressible flow. This may hinder the applicability of the approach for simulations where accurate flow field prediction is insufficient in of itself, and conservation of mass and momentum needs to be guaranteed. Future development should also include models for computing velocity x, y and z components, allowing visualisation of streamlines or possible derivation of parameters such as wall-shear stress. Although we used fully-connected DNNs, other studies have reported the use of long short-term memory (LSTM) networks or specialised architectures such as PointNet to build CFD-based ML models [9, 31, 32]. LSTM networks in particular may be highly suited towards any ML transient flow applications, due to their inherent ability to learn temporal sequences of data. Other architectures such as probabilistic DNNs which output uncertainty intervals during inference should also be explored [31, 33, 34]. Additionally, alternative methods for sampling synthetic data (e.g. Latin hypercube sampling) may produce a more diverse dataset for model training. This may prevent the occurrence of outliers, such as the worst pressure case in the test-set (MNAE_S_=23.6%).

### Potential clinical utility

We believe that the fast computation of haemodynamics using our method has multiple clinical uses. However, this is a proof-of-concept study and further improvements are required prior to any clinical validation (particularly the inclusion of patient-specific boundary conditions and time varying flow fields—see limitations). Nevertheless, if this could be achieved we envisage several clinical uses, such as for supporting the identification of patients who need an intervention and predicting the outcome. Specific to our population, several studies have shown that CFD can be used to evaluate abnormal haemodynamics (particularly during stress) and predict normalisation of haemodynamics after stenting of coarctation. However, this approach is rarely used in the clinical environment because it is so time consuming. We believe our approach could be extrapolated to evaluate stress haemodynamics by simulating each training dataset case under elevated cardiac stress conditions. A second application could involve a fast and automatic pipeline to predict post-stenting haemodynamics in aortas. This could be implemented in a two-step solution using: (i) a surrogate finite-element model for predicting an ideal post-op aortic shape following stenting, and (ii) a surrogate CFD model for predicting haemodynamics on the post-op aortic shape (following the approach we present in this study). Use of such models would bring forth a new level of precision medicine that is currently lacking in congenital heart disease.

## Limitations

In order to translate our DNN-based CFD approach to clinics, there are two main modelling limitations which need to be bypassed. The first is related to the loss in surface accuracy when using SSM representations of aortic shapes. The second is the current simplicity of the CFD approach, which needs to be further developed in order to generate more meaningful DNN training data.

### Shape parameterisation

It has been seen in previous studies that aortic CFD flow fields are highly sensitive to geometric and topological variation [35, 36]. For this reason, using shape vectors that are accurate descriptors of the aortic surfaces is of critical importance. In the future, the relationship between the SSM registration error and the resultant CFD flow fields should be further investigated. Where possible, augmentations to the shape vector should be trialled in order to see if DNN prediction errors for real subjects can be reduced. This may require including additional shape information as inputs in the DNN models to act as a form of regularisation, such as a registration error or a geometric feature (e.g. centreline diameters). Such complimentary shape descriptors may be used to better inform the network of important features not fully captured by the SSM shape vector alone. Additionally, multiple SSMs based upon templates other than the mean aortic shape could be generated. These would enable closer non-rigid registration for unique cases where the target aorta deviates significantly from the mean shape. Recently, Wiputra et al. demonstrated methods for augmenting SSMs to enable the inclusion of head and neck vessel geometry within the aortic shape parameterisation, while retaining high accuracy with low-dimensional PCA vectors [37]. A similar approach may be employed in future studies in order to be able to fully describe the patient-specific aorta with head and neck vessels. Of course, a simple initial improvement could be made by adding more subjects to our SSM to introduce more variability in the population.

### Computational fluid dynamics

In this study, a simplified CFD pipeline was chosen in order to easily automate and ensure convergence for numerous simulations (n = 3,000). A standard CFD solver set-up was used, assuming steady-state conditions and incompressible flow. In order to account for the pulsatility involved in aortic flow, a transient solver may be better suited to real-life applications. It was also assumed that the flow through the great arteries at peak systole was laminar [38]. In the future, the Reynolds number may be computed for individual cases to allow for the inclusion of turbulence modelling where necessary. However, for complex morphologies (such as CoA), Reynolds number has been seen to be an inconsistent measure of turbulence [39]. Feiger et al. proposed an alternative solution to turbulence modelling when performing CFD on a large scale, which involves meshing the domain with extremely high numbers of nodes [10].

Boundary conditions selected included a fixed, flat velocity inlet and zero pressure outlet condition for all cases. However, idealised inlet velocity profiles (flat, parabolic etc.) have been shown to be ineffective for producing clinically relevant data [40, 41]. Thamsen et al. showed that a synthetic aortic population could be created with realistic accompanying 4D MRI-derived vector flow profiles [42]. In the future, a similar approach could be taken, allowing for an additional velocity vector field input parameter into the ML model. Alternatively, a more accessible approach could involve the use of a parabolic velocity inlet condition and a patient-specific unsteady flow profile (derived from phase-contrast MRI) in conjunction with a transient solver for resolving the peak systolic flow field. In this study, it was decided to omit the head and neck vessels from the CFD model for simplicity, however this would be required when aiming to simulate realistic patient-specific aortic haemodynamics [36]. Indeed, Wiputra et al. showed that accurate modelling of the head and neck vessels is necessary for capturing local flow features in the arch and producing realistic downstream fluid flow forces [37]. Thus, the inclusion of head and neck vessels along with lumped parameter outlet models such as Windkessel models should be explored in the future [43, 44], as modelling downstream resistance has been seen to produce more clinically meaningful results [45–47].

## Conclusion

In this proof-of-concept study, we have proposed a pipeline for building ML-based models to perform repetitive vessel-based CFD tasks. Generation of synthetic aortic training data by means of shape modelling allowed ML techniques to be used, even where data scarcity is an issue (n = 67). Point correspondence was maintained between subject meshes in order to enable PCA. Our ML models were able to compute pressure and velocity flow fields much more rapidly (4,000x) than traditional CFD solvers, without large computational requirements or simulation setup. Comparison between predicted and ground truth test cases revealed good overall performance. Testing on prospective cases revealed that shape registration errors could produce misleading flow fields which deviated significantly from the ‘real’ CFD result, even in the presence of low ML errors. The approach described in this study is shape-driven and is applicable to any vascular structure which can be segmented from medical images. In the future, the models should be improved, so they can perform inference on prospective data from real patients. The requirements for this are two-fold; improving the accuracy of the shape representation methods while incorporating the head and neck vessels, and using a more realistic CFD pipeline for generating training data with the inclusion of patient-specific boundary conditions. Comparison of the ML models against clinically acquired data (such as catheter-based pressure drops) should be also performed in the future for validation purposes.

## Supporting information

S1 Fig Volume mesh deformation: Mesh skewness. An example of template volume mesh deformation when generating the volume mesh for a new subject is shown.
(EPS)

S2 Fig CFD sensitivity analysis. A test aortic shape with a sharp arch angulation was chosen to be used to perform a mesh sensitivity study.
(EPS)

S3 Fig Best and worst PCA errors. PCA reconstructions in the test cohort (n = 200) were performed to assess the level of information loss due to dimensionality reduction.
(EPS)

S4 Fig The final pressure and velocity DNN architectures. (EPS)

S5 Fig Regional Bland-Altman analysis. This was conducted on three different regions of the aorta (ascending, transverse arch, descending).
(EPS)

S6 Fig PCA projections of real cases. All ten new cases had their first 35 shape mode scores plotted against the range of the original dataset scores.
(EPS)
